# A Stable Anti-Fouling Coating on PVDF Membrane Constructed of Polyphenol Tannic Acid, Polyethyleneimine and Metal Ion

**DOI:** 10.3390/polym11121975

**Published:** 2019-12-01

**Authors:** Lili Wu, Qiuhu Lin, Cong Liu, Wanyu Chen

**Affiliations:** School of Materials Science and Engineering, Wuhan University of Technology, Wuhan 430070, China; polym_wl@whut.edu.cn (L.W.); 15551719435@163.com (Q.L.); lc_ibelic@163.com (C.L.)

**Keywords:** surface modification, metal ion, anti-fouling, retention rate

## Abstract

A hydrophilic and anti-fouling coating layer was constructed on a polyvinylidene fluoride (PVDF) microfiltration membrane by a novel surface modification method. The pristine membrane was firstly coated by (3-chloropropyl) trimethoxysilane/polyethyleneimine and tannic acid. Then, the metal ion was induced on the coating layer to coordinate with tannic acid and polyethyleneimine, forming a more stable and hydrophilic coating on the surface. The membrane’s surface morphology and chemical element analysis showed that the Tannic acid/ polyethyleneimine (TA/PEI) coating layer was denser and had more stability after the addition of metal ions, and this may be due to the coordination bond formed between the TA/PEI coating and metal ions. The results of the water contact angle and pure water flux measurements showed that the hydrophilicity and wettability of the modified membranes were improved obviously after introducing the metal ion layers. The anti-fouling performance and stability of the modified membrane were also characterized by the underwater oil contact angle (OCA), the separation efficiency, and the contact angle variation value for before and after the rinsing experiment. The modified membrane showed obvious stability and antifouling. Moreover, the retention rate of some composite membranes could reach 99.6%.

## 1. Introduction

Water resources have been overexploited and severely polluted in recent decades due to the population explosion, chemical pollutant emissions, and low reuse rate of domestic sewage [1]. To protect the water on our planet, an efficient and generally accepted strategy is urgently needed for all organizations involved because water resources, such as freshwater lakes, underground rivers, and wetlands, are decreasing [2]. With the persistent research of scientists, membrane science and technology play an increasingly important role in the water treatment fields, which are seawater desalination, ion absorption from water, oil and protein resistance, and gas purification [3]. However, some hydrophobic membranes, which are polluted easily and have short life, cannot address those problems of the purification of domestic sewage, industrial wastewater, and biochemical pollution. Many methods have been developed, for example physical blending [4,5,6], polymeric and surface chemical grating [7,8], surface chemical treatment [9,10], and surface coating [11,12,13,14,15]. Compared with those other methods, surface coating has characteristics such as high efficiency, simple operation, and relatively low cost. The progress of surface modification was accelerated since mussel inspired surface chemistry was studied by Lee et al. in 2007 [16]. Polydopamine has two important characteristics, high catechol (3,4-dihydroxybenzene) content and high primary and secondary amine content [17]. Spontaneous deposition of a polydopamine coating occurs during the formation of polydopamine, and this primary coating can be used directly or used as a “primer” for immobilization of the secondary coating. The secondary coating is highly tailorable in composition and properties, so giving rise to the immense versatility and wide applications enjoyed by polydopamine coatings [18]. Many research works have been done on surface modification via the physical chemistry process of dopamine [19,20,21,22,23,24,25], but dopamine is so expensive and difficult to produce for industry that the research work might not transform into applications for water treatment. In order to solve this problem, tannic acid, which has a similar molecular structure as dopamine, is being studied by more and more scientists in recent years and is considered a potential material for surface modification applications.

Tannic acid has hydrophilicity and can be oxidized under weak alkaline conditions to carry out self-polymerization. During this process, many free radicals would form and react with that of the substrates by covalent and noncovalent bonds. Tannic acid is a hydrogen bond donator, as well as acceptor for producing hydrogen with various substrates [26]. Polyethyleneimine is a highly basic and positively charged aliphatic polymer, containing primary, secondary, and tertiary amino groups in a 1:2:1 ratio. Therefore, every third atom of the polymeric backbone is an amino nitrogen that may undergo protonation [27,28]. Tannic acid can form a crosslinking network with polyethyleneimine (PEI) via Michael addition or Schiff base reaction [29], hence strengthening the force between the crosslinking network and the substrates. At the same time, the addition of polyethyleneimine can facilitate the deposition of PEI, which provides an amine group to react with tannic acid [30], forming hydrophilic coating layers on the surface of membranes and reducing the deposition time and membrane pore blockage [19]. In addition, tannic acid and PEI can both react with metal ions by coordination to construct a three-dimensional crosslinking network, which is stable and has anti-fouling properties [31,32,33,34].

In order to improve the stability between the membrane surface and polyethyleneimine, a chemical substance needs to be introduced. The silane coupling agent hydrolyzes to generate silanol groups, then forms oligosiloxane by dehydration and condensation after contact with moisture in the air, which is favorable to enhance the crosslinking. Some silane coupling agents possess functional groups that can react with PEI, thus promoting its crosslinking [35,36,37]. Yang et al. used the amine derivatives to react with p-xylylene dichloride (XDC) via quaternization [38]; therefore, PEI can react with (3-chloropropyl) trimethoxysilane (CTS) using the same chemical mechanism. CTS has a similar structure to the silane coupling agent, so it can crosslink with PEI to form a stable network.

In this study, a stable and anti-fouling polyvinylidene fluoride (PVDF) microfiltration membrane was fabricated by surface coating, using tannic acid (TA), PEI, and metal ions as hydrophilic modifiers. Firstly, the pristine PVDF microfiltration membranes were coated by PEI/CTS coating, then decorated by TA. After that, the membranes were further modified by metal ions (Zr^4+^, Fe^3+^, Al^3+^, Cu^2+^, Zn^2+^, or Mn^2+^) to obtain the final modified PVDF microfiltration membranes that were hydrophilic, stable, and resistant to oil. Moreover, the influence of the modification time of every metal ion on the modified membrane’s properties was studied.

## 2. Materials and Methods

### 2.1. Materials

Tannic acid (TA), polyethyleneimine (PEI, *M*_w_ = 600 Da), and (3-chloropropyl) trimethoxysilane (CTS) were purchased from Aladdin (Aladdin Industrial Corporation, Shanghai, China). Zr(SO_4_)_2_, FeCl_3_, Al_2_(SO_4_)_3_·18H_2_O, Cu(NO_3_)_2_·3H_2_O, Zn(NO_3_)_2_·6H_2_O, and MnSO_4_ were bought from Sinopharm Chemical Reagent Co., Ltd. (Shanghai, China). The PVDF microfiltration membranes (MF, 0.22 µm) were obtained from Membrane Solutions (Nantong, China). Deionized water was prepared from a deionized water generator system (RX-108, XINRUI, Tengzhou xinrui analytical instrument Co. Ltd., Tengzhou, China). Isopropyl alcohol, tris(hydroxymethyl)aminomethane (Tris), hydrochloric acid (HCl), toluene, Tween-80, and chloroform were purchased from Sinopharm Chemical Reagent Co., Ltd. (Shanghai, China). In this work, all chemical reagents were analytical grade unless specified otherwise, which were used without further purification.

### 2.2. Preparation of TA/PEI/M Modified PVDF MF Membranes

**Figure 1:**
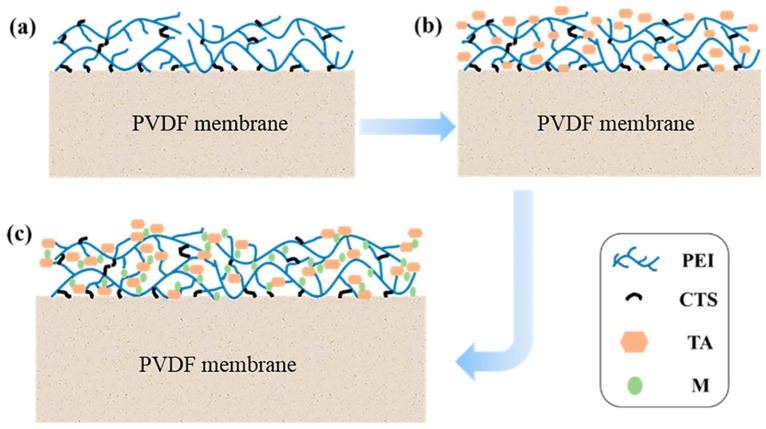
The modification process of the PVDF membrane: (**a**) the membrane coated with PEI/(3-chloropropyl) trimethoxysilane (CTS); (**b**) the membrane coated with PEI/CTS-TA; (**c**) the membrane coated with PEI/CTS-tannic acid (TA)-M (metal ion).

The preparation of TA/PEI modified PVDF MF membranes was reported in our previous work [39]. In brief, the pristine PVDF MF membranes were pretreated by isopropanol for 0.5 h and then immersed in deionized water for another 0.5 h to clear their pores. After that, the pretreated membranes were soaked in the PEI/CTS solution, which was prepared by 20 mg PEI, 20 mg CTS, and 100 mL deionized water for 10 h at 25 °C. Next, the membranes were washed with deionized water several times and immersed in 100 mL Tris-HCl buffer (pH = 7.8) that contained 20 mg TA for 12 h at 25 °C. Finally, the membranes were immersed in 100 mL 0.2 g/L metal ion solution for a certain time (1 min, 3 min, 5 min, 10 min 15 min, or 30 min) at 25 °C, obtaining the tannic acid, polyethyleneimine and mental ions (TA/PEI/M) composite modified PVDF MF membranes (x-mM, where “x” is the soaking time, “m” means the membrane, and “M” is the metal ion). The modification process is shown in Figure 1.

### 2.3. Physicochemical Properties of Modified PVDF MF Membranes

The surface chemical elements of the modified PVDF MF membranes were characterized by X-ray photoelectron spectroscopy (XPS, ESCALAB250Xi, Thermo-Fisher, Waltham, MA, USA), which used Al-Kα as the radiation source. The membrane surface morphology was observed by field-emission scanning electron microscope (FESEM, Zeiss Ultra Plus, Zeiss, Germany) with an accelerating voltage of 5.0 kV.

### 2.4. Hydrophilicity and Oleophobicity of Modified PVDF MF Membranes

The hydrophilicity of the membranes was characterized by the water contact angle (WCA) and pure water flux (WF). The oleophobicity of uncoated and coated membranes was characterized by the underwater oil contact angle (OCA) and flux recovery ratio (FRR). The WCA and OCA were measured by a contact angle test system (JC2000C, Zhongchen, Shanghai zhongchen digital technology equipment Co. Ltd., Shnaghai, China). In addition, the emulsion flux of the membranes was also tested to study the membrane separation ability. The emulsion was made by mixing toluene and water (1:99, *v*/*v*) with the addition of 0.02 mg Tween-80 per milliliter of emulsion under a high stirring speed for at least 4 h. The emulsion flux and water flux were done by a vacuum filter system (JINTENG, Tianjin jinteng technology Co. Ltd., Tianjin, China) at a stable pressure (0.1 MPa) and at room temperature, and the average value of three measurements of every sample was taken. Moreover, the rejection for the emulsion was characterized by the UV-visible absorption spectrum (UV-Vis). The flux (F), FRR, and emulsion rejection ratio (*R*_j_) were calculated using Equations (1)–(3), respectively:

(1) $$F=\frac{V}{A\Delta t}$$

(2) $$\;\mathrm{FRR}=\frac{F_{w_{2}}}{F_{w_{1}}}\times 100\%\;$$

(3) $$\;R_{j}=\left(1-\frac{C_{P}}{C_{0}}\right)\times 100\%$$

where *V*, *A*, and Δt represent the permeate volume (L), the effective membrane area (m^2^), and the filtration time (h), respectively. *F*w_1_ and *F*w_2_ represent the pure water flux before and after the emulsion filtration, respectively. *C*_p_ and *C*_0_ represent the concentration of the permeate solution and original solution, respectively. The concentration was measured by a UV–Vis spectrophotometer at 261 nm for toluene.

### 2.5. Hydrophilic Stability of Modified PVDF MF Membranes

The hydrophilic stability of the membranes was measured by the long term rinsing experiment in pure water for seven days, namely the membranes were fixed to the wall of a beaker with a magnetic stirrer, then rinsed by the flow of water. The WCA and pure water flux of the pristine and modified membranes before and after the rinsing experiment were tested. Moreover, the contact angle variation value ($\Delta_{CA}$) and contact angle recovery ratio (CR) were used firstly to study the stability of the membranes, which were calculated by Equations (4) and (5):

(4) $$\Delta_{CA}=\;CA_{0}-\;CA_{1}$$

(5) $$\mathrm{CR}=\frac{CA_{1}-CA_{2}}{CA_{1}-CA_{0}}$$

where CA_0_, CA_1_, and CA_2_ represent the contact angle of the modified membrane, the membrane after the pure water flux test, and the recovery membrane, which was soaked in the pure water for 72 h after the pure water flux test.

## 3. Results and Discussion

### 3.1. Surface Characterization of the Chemical Elements

**Figure 2:**
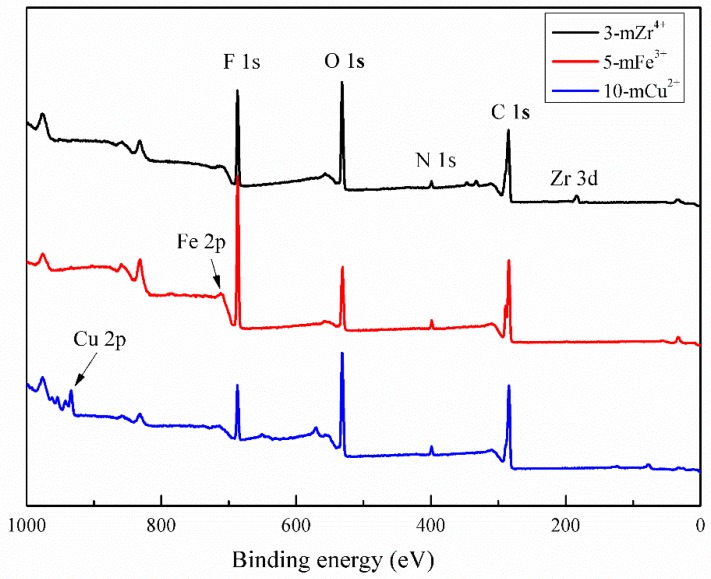
XPS spectra of the 3-mZr^4+^, 5-mFe^3+^, and 10-mCu^2+^ membrane.

Figure 2 shows the XPS spectra of the TA/PEI/M modified PVDF MF membranes. There were obvious new signals, which represented the Zr 3d, Fe 2p, and Cu 2p peak in the spectra of the 3-mZr^4+^, 5-mFe^3+^, and 10-mCu^2+^ membranes, respectively. Moreover, the O and N signal could also be observed, indicating that TA and PEI were coated on the membrane surface successfully. The chemical composition of the modified membranes is shown in Table 1. The O/N ratios of the modified membranes were different. The O/N ratio of 3-mZr^4+^ and 10-mCu^2+^ was relatively large, which was due to the addition of acid ions such as SO_4_^2−^ from Zr(SO_4_)_2_ and NO_3_^−^ from Cu(NO_3_)_2_; however, the use of FeCl_3_ did not provide acid ions, so that the O/N ratio of the 5-mFe^3+^ membrane was the minimum.

### 3.2. Surface Morphologies of the TA/PEI/M Modified Membranes

**Figure 3:**
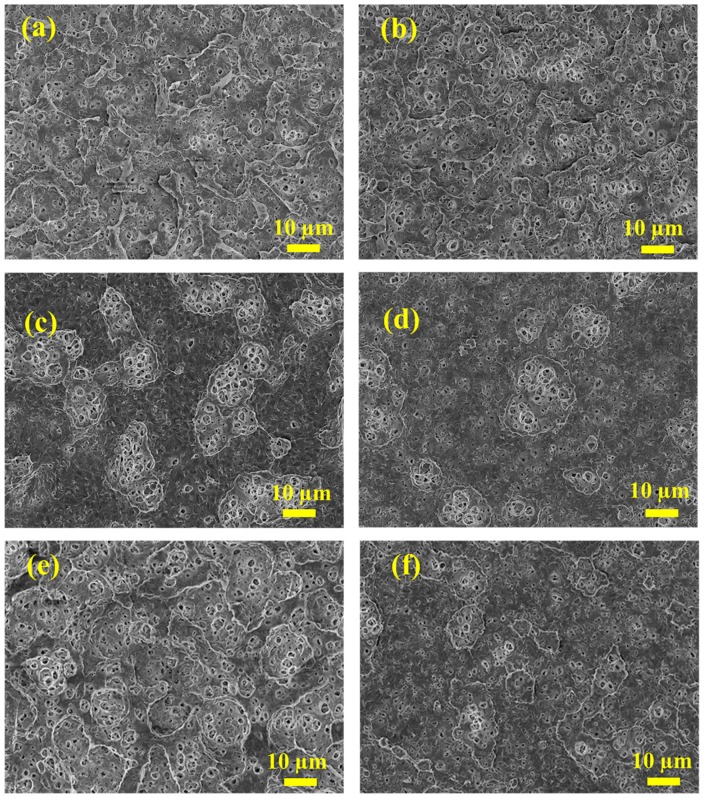
FESEM images of TA/PEI/M modified PVDF microfiltration (MF) membranes. (**a**,**b**,**c**,**d**,**e**,**f**) The 3-mZr^4+^, 5-mFe^3+^, 3-mAl^3+^, 10-mCu^2+^, 3-mZn^2+^, and 3-mMn^2+^ membranes, respectively.

The surface morphologies of pristine and modified PVDF MF membranes are given in Figure S1 (from the supplementary materials) and Figure 3, respectively. Compared with the pristine membrane, which possessed many bumps on the surface, the TA/PEI modified membrane was obviously coated by the coating layers formed by TA and PEI to cover the bumps or around them, after the addition of metal ions, the coating layers were connected to each other and more obvious around the bumps, such as the coating layers of the 3-mZr^4+^, 5-mFe^3+^, and 3m-Zn^2+^ membranes. The coating layer area on the surface of the 3-mAl^3+^, 10-mCu^2+^, and 3-mMn^2+^ membranes was thicker, which could be explained by two facts. First, the Cu^2+^ with strong oxidizing ability could further improve the oxidation degree of TA. Secondly, the structure of the layers was changed by the metal ions, which could coordinate with TA and PEI, increasing their crosslinking and making the coating layers more compact [40]. These results indicate that the introduction of a hydrophilic modifier (TA/PEI or TA/PEI/M) could influence the morphology of the membranes.

### 3.3. Hydrophilicity of the TA/PEI/M Modified Membranes

**Figure 4:**
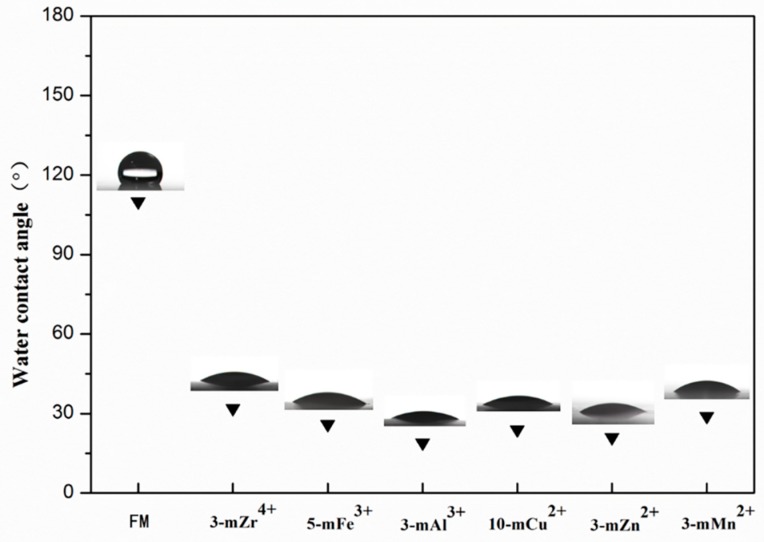
The WCA of the pristine PVDF membrane (FM) and TA/PEI/M modified PVDF MF membranes.

**Figure 5:**
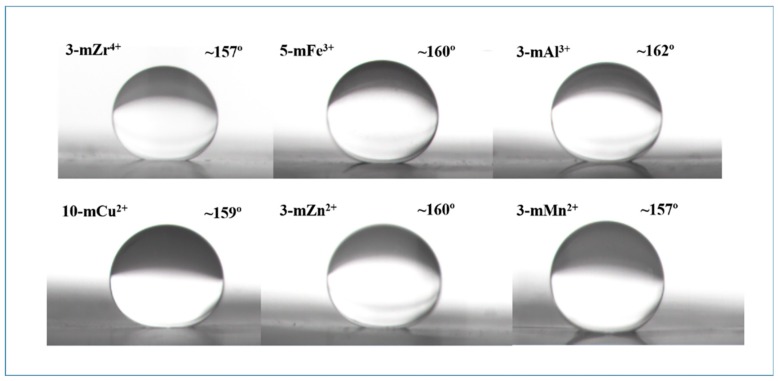
The oil contact angle (OCA) of the TA/PEI/M modified PVDF MF membranes.

The influence of the modification time of metal ions on the hydrophilicity of the modified membranes is shown in Figures S2–S13 (from the supplementary materials). The hydrophilicity of TA/PEI/M modified PVDF MF membranes improved greatly, and the largest pure water flux was 13,028 L/m^2^·h (10-mCu^2+^), while the smallest water contact angle was 19° (3m-Al^3+^). On the one hand, as the metal ions had different oxidation states, forming different hydrophilic coating layers that had various structures, especially the metal ions with a higher oxidation state could induce the polymerization of TA, increasing its crosslinking with PEI on the surface of the membranes. On the other hand, the element content of the modified membrane surface may also influence the hydrophilicity of the membranes, as seen by the XPS and contact angle analysis. The contact angle images of modified membranes are given in Figure 4 and Figure 5. Compared with the pristine membrane, the WCA of TA/PEI/M modified membranes decreased obviously, and the OCA of the modified membranes was larger than 157°; the OCA of the pristine PVDF MF membrane was only 140°, and all the results demonstrated that the hydrophilicity and oleophobicity of the TA/PEI/M modified membranes were enhanced. Owning to the coating of the hydrophilic layers formed, the surface roughness of the TA/PEI/M modified membrane increased obviously. With the change of the membrane surface roughness, the surface capillary action and solid-liquid-gas three-phase balance of the membrane would be directly impacted. According to the mechanism of surface roughness, the rougher the surface is, the more hydrophilic the surface. As a result, the surface roughness of the modified membranes increased, leading to the membrane surface being more hydrophilic and oleophobic.

### 3.4. Anti-Fouling Performance of TA/PEI/M Modified Membranes

**Figure 6:**
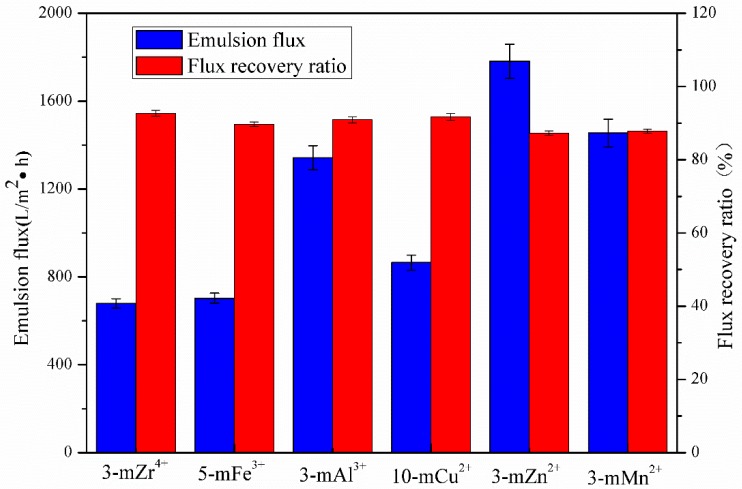
Emulsion flux and flux recovery ratio of TA/PEI/M modified membranes.

**Figure 7:**
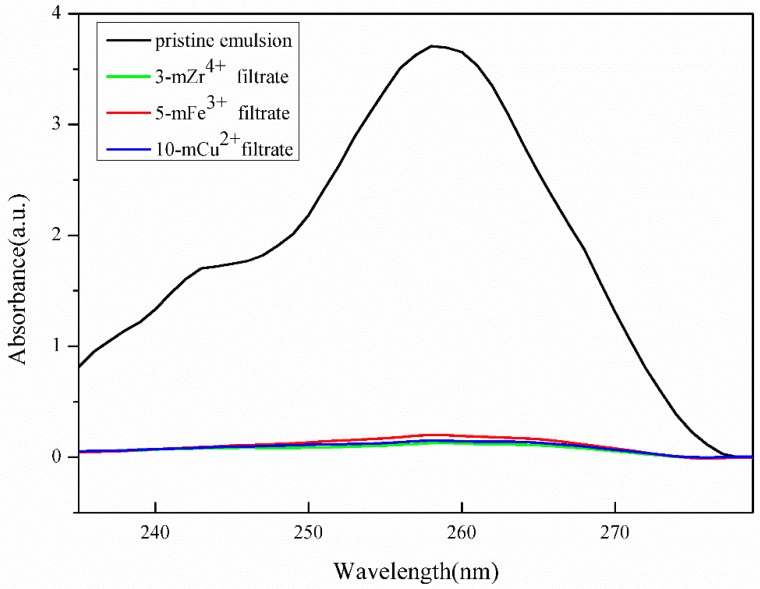
The UV-Vis spectrum of the pristine emulsion and the filtrate of 3-mZr^4+^, 5-mFe^3+^, and 10-mCu^2+^ membranes.

**Figure 8:**
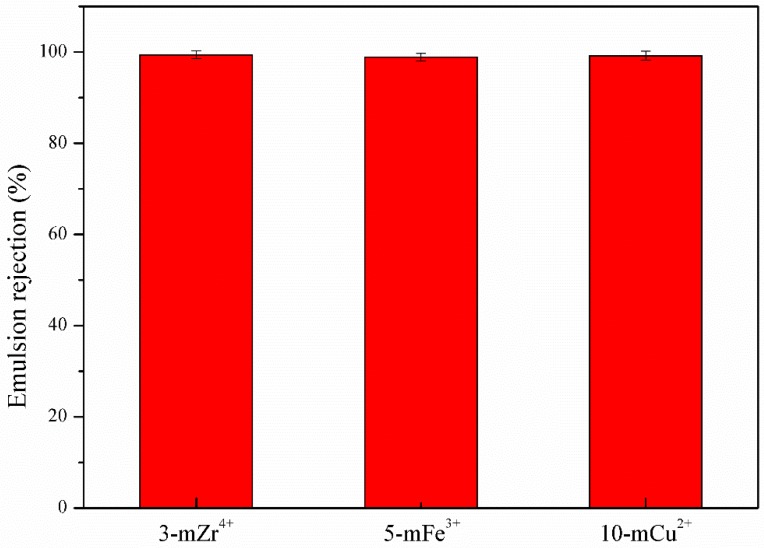
Emulsion rejection of 3-mZr^4+^, 5-mFe^3+^, and 10-mCu^2+^ membranes.

The emulsion flux and flux recovery ratio of the modified membranes are shown in Figure 6. Because the metal ions with a higher oxidation state, such as Zr^4+^, Fe^3+^, and Cu^2+^, could induce the polymerization of TA to compact the coating layers, the pores of the membranes were covered by those hydrophilic coating layers, and it was difficult for the emulsion to pass through them. The FRR of the 3-mZr^4+^, 5-mFe^3+^, and 10-mCu^2+^ membranes was 92.7%, 89.7%, and 91.7%, respectively, and the FRR of the 3-mAl^3+^, 3-mZn^2+^, and 3-mMn^2+^ membranes was 90.9%, 87.3%, and 87.8%, respectively. Because the surface roughness of the 3-mAl^3+^, 3-mZn^2+^, and 3-mMn^2+^ modified membranes was relatively greater by SEM analysis, which could increase the contaminated surface area, the FRR of the 3-mAl^3+^, 3-mZn^2+^, and 3-mMn^2+^ membranes was relatively lower. The resistance of the 3-mZr^4+^, 5-mFe^3+^, and 10-mCu^2+^ membranes is given in Figure 7 and Figure 8; the peak of toluene nearly disappeared, improving the outstanding anti-fouling performance of the modified membranes, and the emulsion rejection ratio resistance of all modified membranes for the toluene emulsion was approximately 99%. The emulsion rejection of the 3-mZr^4+^ membrane was 99.6%. This was due to zr^4+^ and Fe^3+^ being hard acids and Cu^2+^ being a junction acid, while the primary amine and secondary amines in polyethyleneimine are hard bases. According to the Pearson hard-soft-acid-base (HSAB) theory, those metal ions can coordinate with PEI and TA to form a complex crosslinking network on the surface of the pristine PVDF membranes, effectively improve the emulsion rejection ratio of the modified membranes.

### 3.5. Stability of TA/PEI/M Modified Membranes

**Figure 9:**
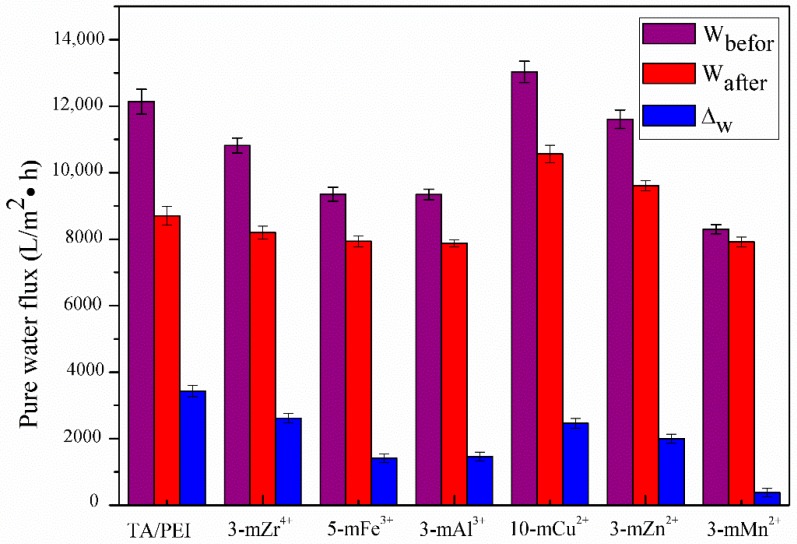
Pure water flux of the TA/PEI/M and TA/PEI modified membranes before and after the rinsing experiment and the pure water flux variation ($\Delta_{W})$.

Figure 9 shows the pure WF of the TA/PEI and TA/PEI/M modified membranes. It can be seen that the 10-mCu^2+^, 3-mZn^2+^, and 3-mZr^4+^ modified membranes had significant improvements compared with the TA/PEI modified membrane. This was due to Cu^2+^, Zn^2+^, and Zr^4+^ having a larger ionic radius (74 pm, 73 pm, and 72 pm, respectively) compared with Fe^3+^, Al^3+^, and Mn^+2^ (50 pm, 55 pm, and 67 pm, respectively), resulting in their (Cu^2+^, Zn^2+^, and Zr^4+^) ability to increase hydrophilicity, while effective reducing the clogging of the pores of the modified membranes. The pure water flux of the 5-mFe^3+^, 3-mAl^3+^, and 3-mMn^2+^ modified membranes slightly decreased due to the blockage of the modified membranes’ pores.

**Figure 10:**
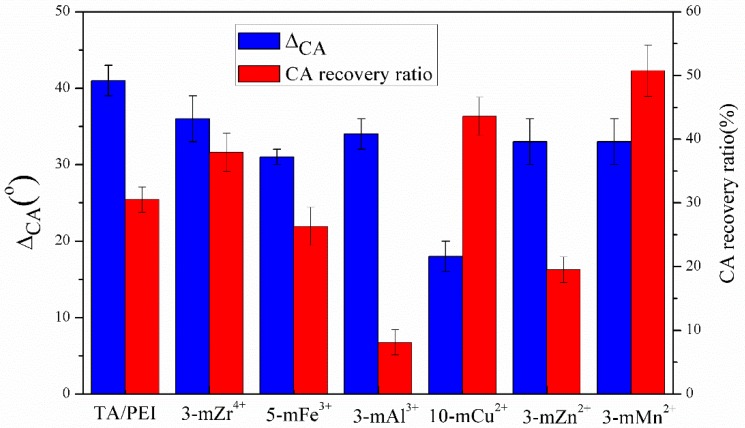
The water contact angle (CA) variation ($\Delta_{CA})$ and CR of the TA/PEI/M and TA/PEI modified membranes.

The variation of pure WF and WCA is shown in Figure 9 and Figure 10, respectively. The pure WF and WCA variation value of the TA/PEI/M modified PVDF MF membranes was obviously lower than the TA/PEI modified membranes, illustrating that the stability of the modified membranes was further strengthened after the addition of the metal ions, which could coordinate with both TA and PEI. The pure WF variation value of the 3-mMn^2+^ membrane was the smallest, and the WCA variation value of the 10-mCu^2+^ membrane was the lowest. As the electron orbits of the metal atoms were different, they had different ways of reacting with TA and PEI. In addition, due to different metal ions having unequal hydration energy, that is by metal cations combined with water releasing energy, this would directly impact the solubility of metal ions in aqueous solution. When the hydration energy increased, the solubility of the coating in aqueous solution and stability of the modified membrane were also enhanced. The coating layers of the TA/PEI/M modified PVDF MF membranes had different morphologies that were significant; therefore, the pure water flux variation ($\Delta_{W})$, $\Delta_{CA}$, and CR of every TA/PEI/M modified membrane were different.

## 4. Conclusions

A super oleophobic PVDF MF membrane was prepared by a simple surface modification method that used TA, PEI, and metal ions. The membrane possessed better stability in pure water when metal ions were applied to coordinate with TA and PEI. The 3-mAl^3+^ membrane showed the minimum WCA (19°) and maximum OCA (162°), and the 10-mCu^2+^ membrane had the maximum water permeability (13,028 L/m^2^·h). In addition, the resistance of TA/PEI/M modified PVDF MF membranes to toluene was approximately 99%, and the FFR of the modified membranes was higher than 87%, displaying their good anti-fouling performance. This study can provide new insight for the field of surface hydrophilic modification, as inexpensive and eco-friendly modifiers such as tannic acid could see more applications in this field.

## Figures and Tables

**Table 1:** The chemical composition of the modified membranes.

Modified Membranes	Composition (at.%)						
	C	O	N	Zr	Fe	Cu	O/N
3-mZr^4+^	68.11	28.19	3.04	0.65	0	0	9.27
5-mFe^3+^	75.78	18.37	3.42	0	2.43	0	5.37
10-mCu^2+^	66.06	27.61	3.68	0	0	2.53	7.50

## Supplementary Materials

The following are available online at https://www.mdpi.com/2073-4360/11/12/1975/s1: Figure S1: FESEM images of the pristine and TA/PEI modified PVDF MF membrane, Figure S2: The influence of modification time of Zr^4+^ on WCA and OCA of the TA/PEI/M modified PVDF MF membranes. Figure S3: The influence of modification time of Zr^4+^ on WF of the TA/PEI/M modified PVDF MF membranes, Figure S4: The influence of modification time of Fe^3+^ on WCA and OCA of the TA/PEI/M modified PVDF MF membranes, Figure S5: The influence of modification time of Fe^3+^ on WF of the TA/PEI/M modified PVDF MF membranes, Figure S6: The influence of modification time of Al^3+^ on WCA and OCA of the TA/PEI/M modified PVDF MF membranes, Figure S7: The influence of modification time of Al^3+^ on WF of the TA/PEI/M modified PVDF MF membranes, Figure S8: The influence of modification time of Cu^2+^ on WCA and OCA of the TA/PEI/M modified PVDF MF membranes, Figure S9: The influence of modification time of Cu^2+^ on WF of the TA/PEI/M modified PVDF MF membranes, Figure S10: The influence of modification time of Zn^2+^ on WCA and OCA of the TA/PEI/M modified PVDF MF membranes, Figure S11: The influence of modification time of Zn^2+^ on WF of the TA/PEI/M modified PVDF MF membranes, Figure S12: The influence of modification time of Mn^2+^ on WCA and OCA of the TA/PEI/M modified PVDF MF membranes, Figure S13: The influence of modification time of Mn^2+^ on WF of the TA/PEI/M modified PVDF MF membranes.
