# Supplementary material for: A Stable Anti-Fouling Coating on PVDF Membrane Constructed of Polyphenol Tannic Acid, Polyethyleneimine and Metal Ion
Source: Polymers (Basel). 2019 Dec 1;11(12):1975. doi: 10.3390/polym11121975 (PMC6960656; doi:10.3390/polym11121975)
Supplement: Supplementary file 1 [file polymers-11-01975-s001.pdf]

polymers

Supplementary Materials

A Stable Anti-Fouling Coating on PVDF Membrane Constructed of Polyphenol Tannic Acid, Polyethyleneimine and Metal Ion

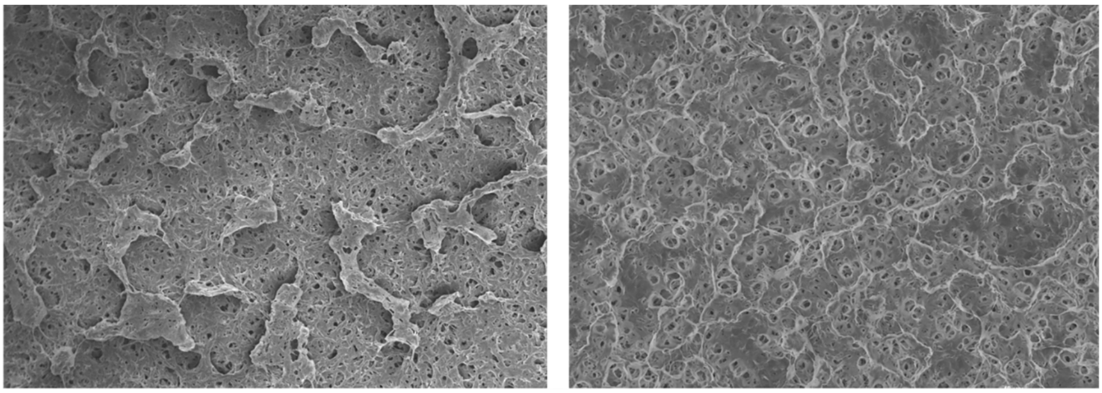

Pristine PVDF MF membrane **Membrane modified by TA/PEI**

Figure S1. FESEM images of the pristine and TA/PEI modified PVDF MF membrane.

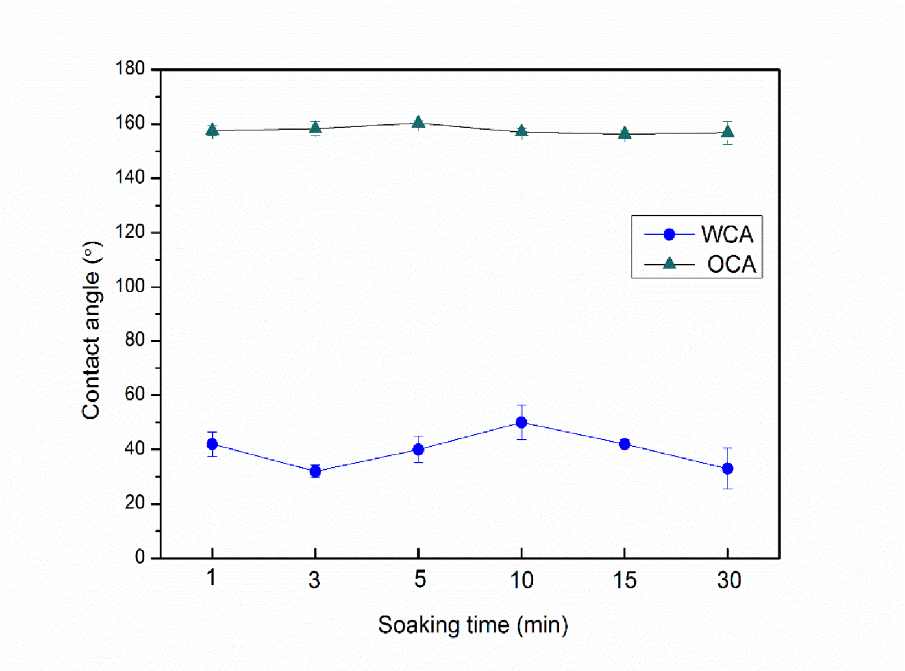

Figure S2. The influence of modification time of Zr^{4+} on WCA and OCA of the TA/PEI/M modified PVDF MF membranes.

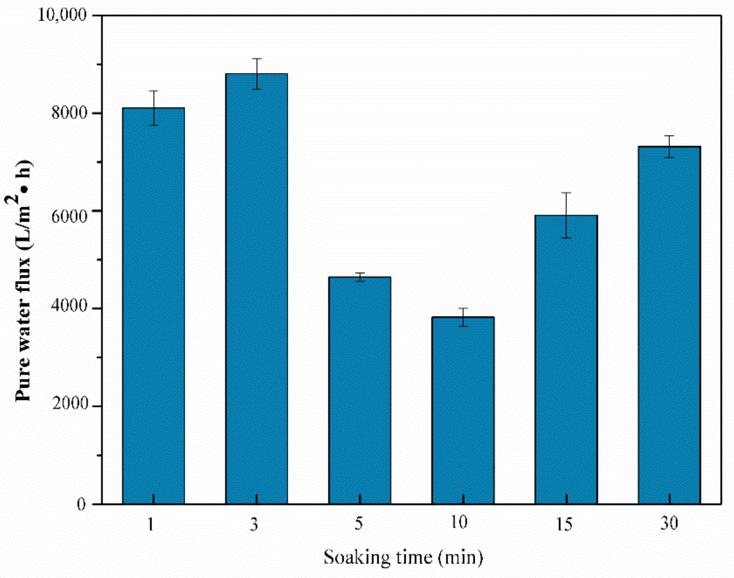

Figure S3. The influence of modification time of Zr^{4+} on WF of the TA/PEI/M modified PVDF MF membranes.

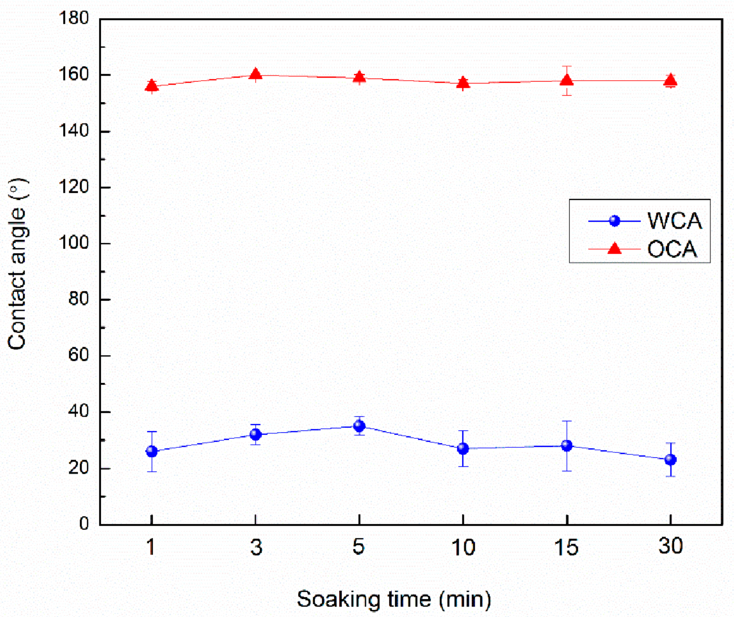

Figure S4. The influence of modification time of Fe^{3+} on WCA and OCA of the TA/PEI/M modified PVDF MF membranes.

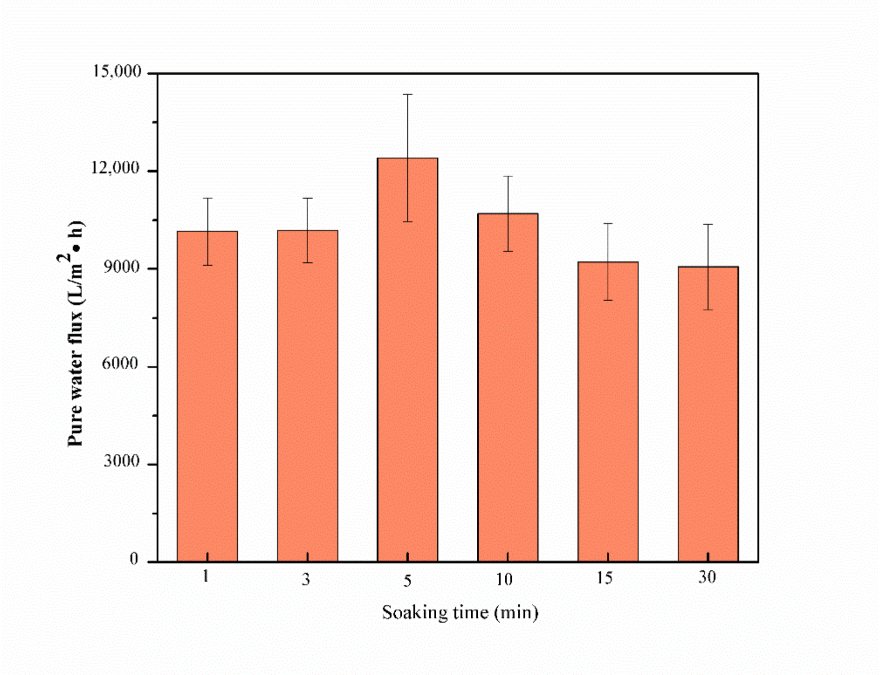

Figure S5. The influence of modification time of Fe^{3+} on WF of the TA/PEI/M modified PVDF MF membranes.

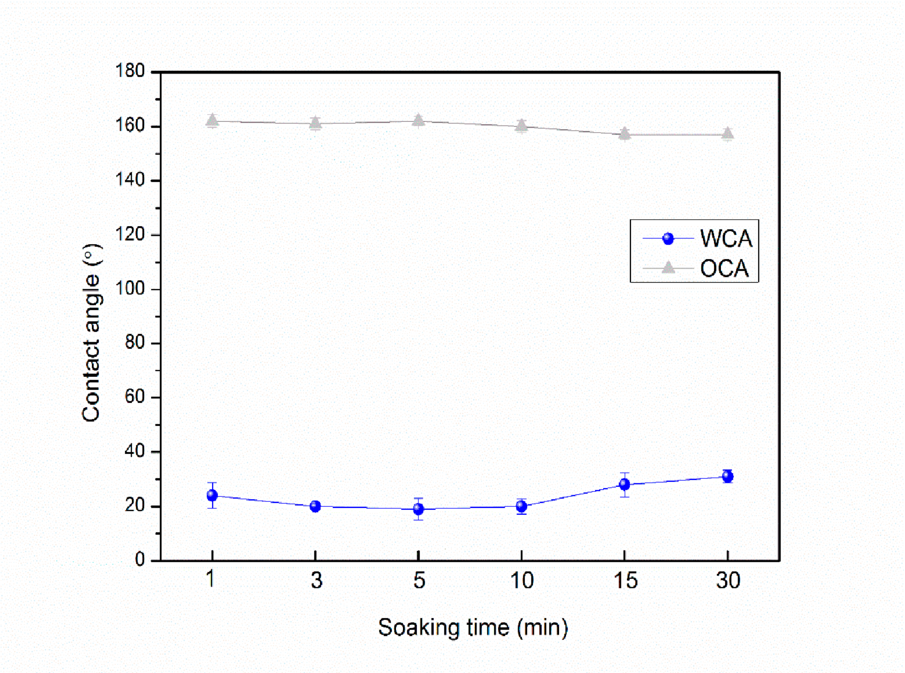

Figure S6. The influence of modification time of Al^{3+} on WCA and OCA of the TA/PEI/M modified PVDF MF membranes.

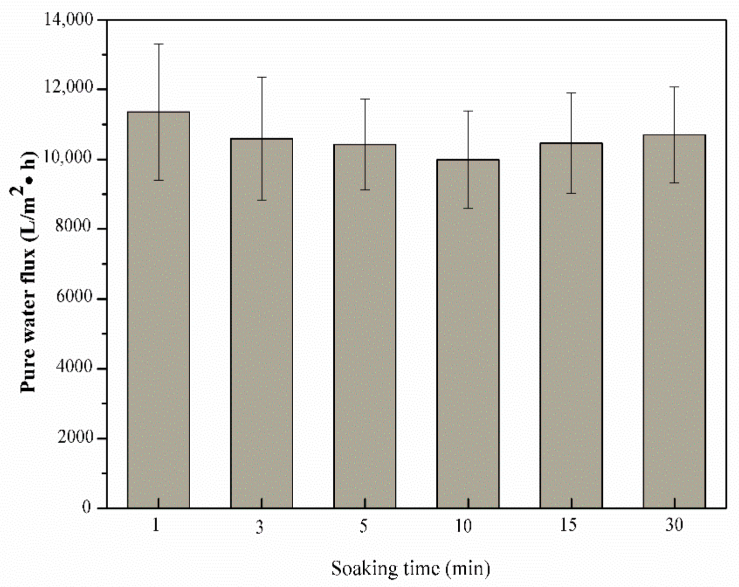

Figure S7. The influence of modification time of Al^{3+} on WF of the TA/PEI/M modified PVDF MF membranes.

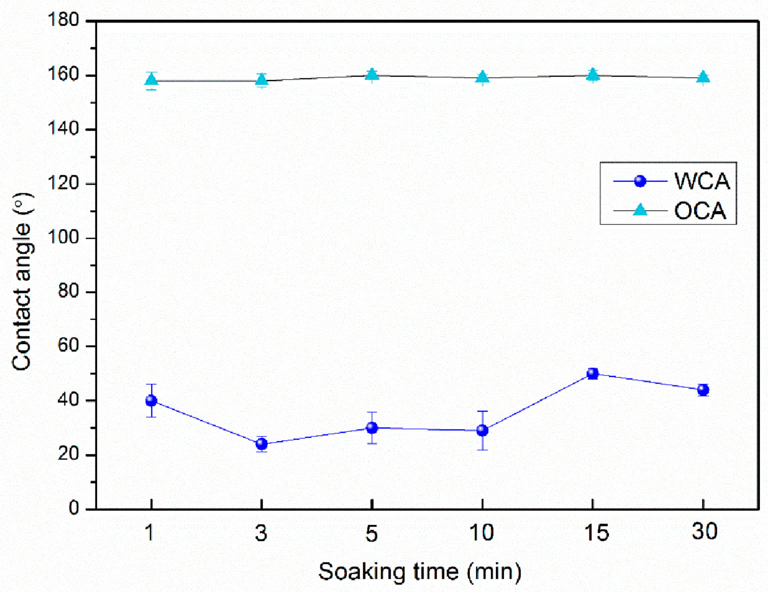

Figure S8. The influence of modification time of Cu^{2+} on WCA and OCA of the TA/PEI/M modified PVDF MF membranes.

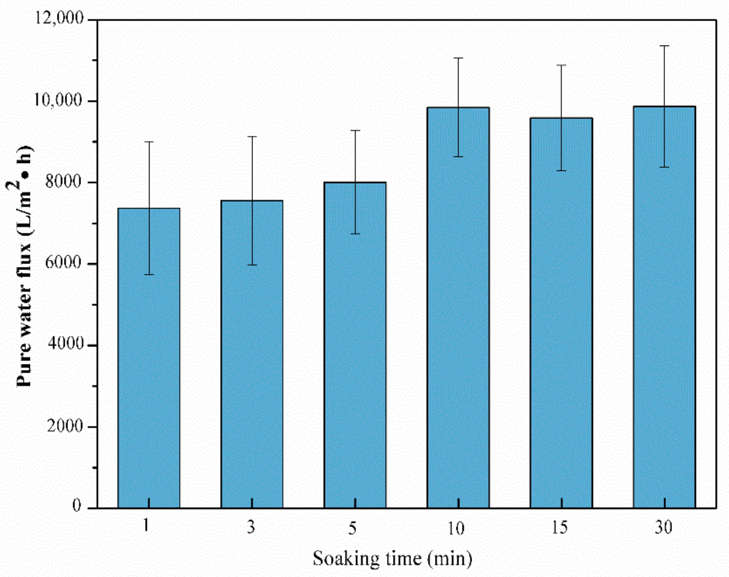

Figure S9. The influence of modification time of Cu^{2+} on WF of the TA/PEI/M modified PVDF MF membranes.

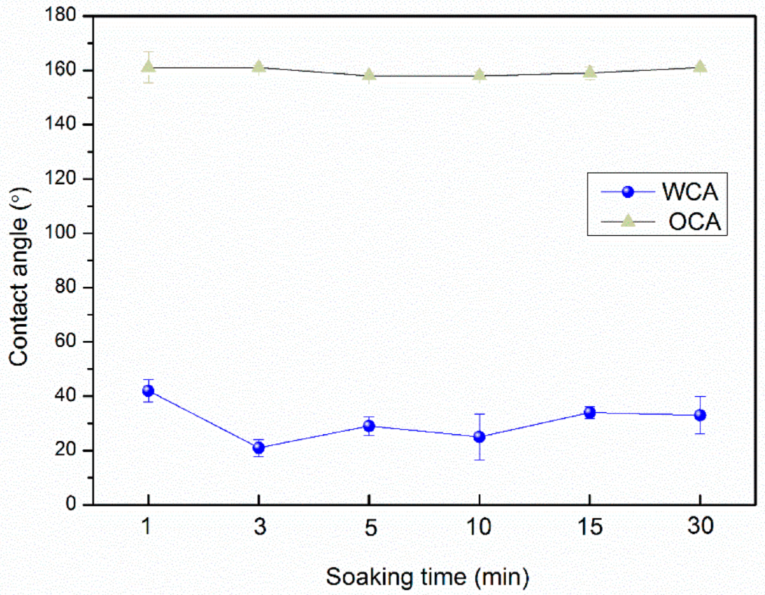

Figure S10. The influence of modification time of Zn^{2+} on WCA and OCA of the TA/PEI/M modified PVDF MF membranes.

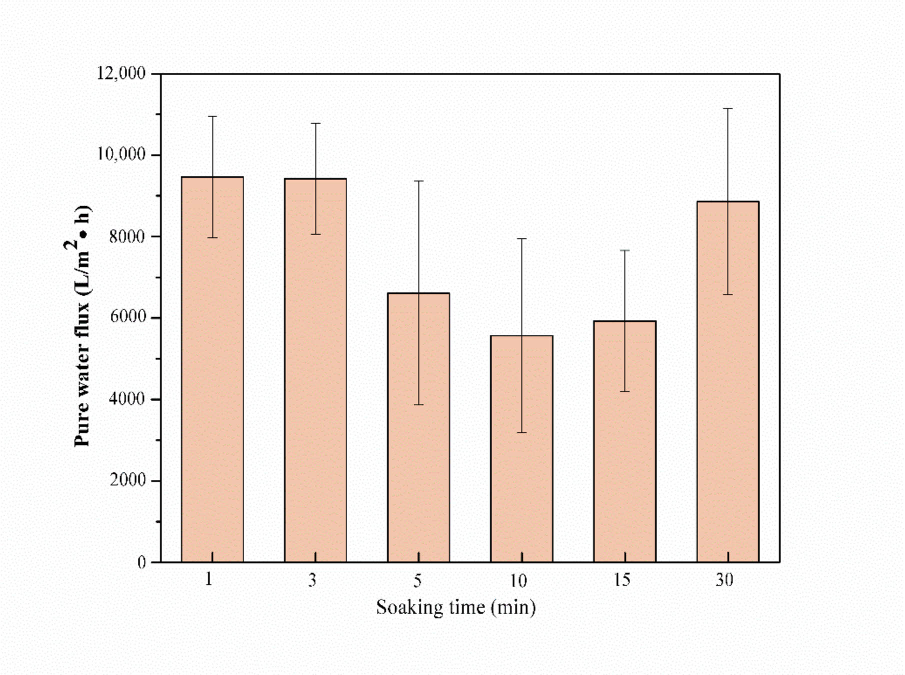

Figure S11. The influence of modification time of Zn^{2+} on WF of the TA/PEI/M modified PVDF MF membranes.

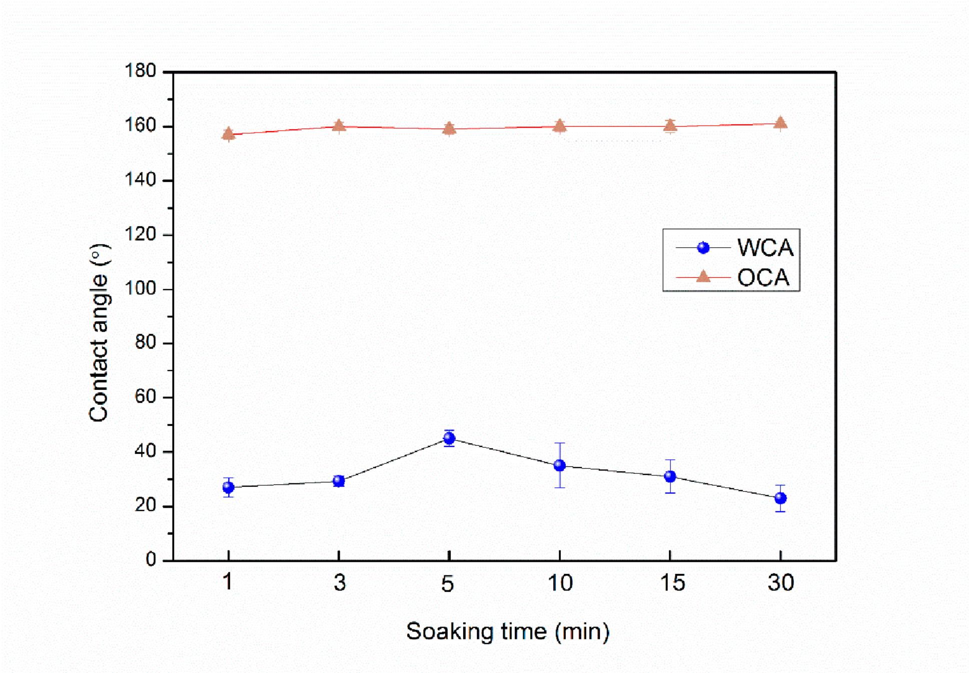

Figure S12. The influence of modification time of Mn^{2+} on WCA and OCA of the TA/PEI/M modified PVDF MF membranes.

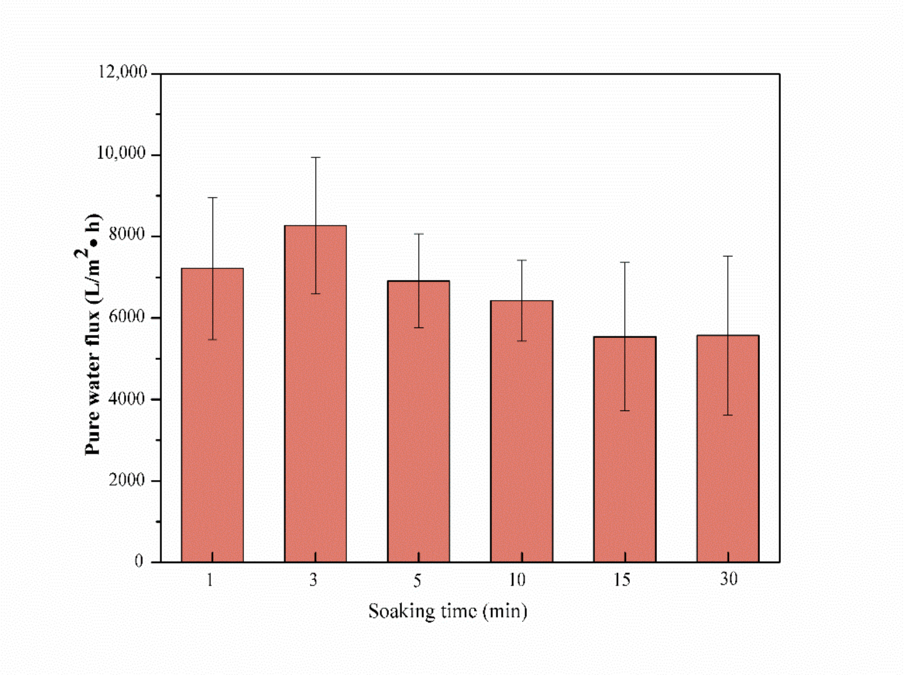

Figure S13. The influence of modification time of Mn^{2+} on WF of the TA/PEI/M modified PVDF MF membranes.
